# Hindgut Duplication in an Infant with Omphalocele–Exstrophy–Imperforate Anus–Spinal Defects (OEIS) Complex

**DOI:** 10.1055/s-0041-1742154

**Published:** 2022-03-10

**Authors:** Timothy F. Tirrell, Farokh R. Demehri, Craig W. Lillehei, Joseph G. Borer, Benjamin C. Warf, Belinda H. Dickie

**Affiliations:** 1 Department of Surgery, Boston Children's Hospital, Boston, Massachusetts, United States; 2 Department of Urology, Boston Children's Hospital, Boston, Massachusetts, United States; 3 Department of Neurosurgery, Boston Children's Hospital, Boston, Massachusetts, United States

**Keywords:** OEIS, cloacal exstrophy, enteric duplication

## Abstract

**Introduction** The congenital anomaly of omphalocele, cloacal exstrophy, imperforate anus, and spinal abnormalities (OEIS complex) is rare but well recognized. Hindgut duplications are also uncommon and are not known to be associated with OEIS. We describe a neonate with OEIS who was found to have fully duplicated blind-ending hindguts.

**Case Report** A premature infant boy with OEIS underwent first-stage closure on day of life 6, which included excision of the omphalocele sac, separation of the cecal plate and bladder halves, tubularization of the cecal plate, hindgut rescue with end colostomy, and joining of the bladder halves. Cecal plate inspection revealed two hindgut structures that descended distally, one descended midline into the pelvis along the sacrum and the second laterally along the left border of the sacrum. Both lumens connected to the cecal plate and had separate mesenteries. In an effort to maximize the colonic mucosal surface area, the hindgut segments were unified through a side-to-side anastomosis, creating a larger caliber hindgut. The cecal plate was tubularized and an end colostomy was created. Bowel function returned and he was discharged home on full enteral feeds.

**Discussion** This case represents a cooccurrence of two extremely rare and complex congenital anomalies. The decision to unify the distinct hindguts into a single lumen was made in an effort to combine the goals of management for both OEIS and alimentary duplications. The hindgut is abnormal in OEIS and should be assessed carefully during repair.

## Introduction

The management of the collection of congenital malformations including omphalocele, cloacal exstrophy, imperforate anus, and spinal abnormalities (OEIS complex) has evolved over the past decades. Once carrying significant mortality risk, 1 patients now have excellent survival rates with appropriate surgical management. 2 3 With separation and internalization of the gastrointestinal and genitourinary tracts, dehydration and electrolyte imbalances can be mitigated. This reconstruction is often performed in stages over several months, with the first stage involving separation of the cecal and bladder plates, tubularization of the cecal plate, and “rescue” of the hindgut to create an end colostomy. At birth, the hindgut is distal to the exstrophic cecal plate, and therefore is not usually in the fecal stream. The hindgut is generally diminutive (<20 cm) 4 and blind ending. At the time of cecal plate separation and tubularization, the hindgut is mobilized and an end colostomy is created, allowing the hindgut to grow and play a critical role in preventing dehydration. 5

Alimentary tract duplications have been described at all locations in the gastrointestinal tract and are found most frequently in the small bowel. Colonic and rectal duplications are relatively uncommon, accounting for approximately 16 and 4% of enteric duplications, respectively. 6 Enteric duplications are more commonly cystic than tubular, and this generality holds true for colonic and rectal duplications. Colonic and rectal duplications are often found in conjunction with urinary tract anomalies, 7 likely owing to their common embryologic origin.

Here, we describe a neonate born with OEIS complex with duplication of the entire hindgut. The duplicated hindguts developed as two distinct structures, with individual mesenteries, and travelled distally along opposite sides of the pelvis. To preserve colonic surface area, we created a longitudinal side-to-side anastomosis between the two structures to create a single functional hindgut, which was brought up to the abdominal wall as an end colostomy. This is a rare combination of two uncommon malformations.

## Case Report

**Fig. 1:**
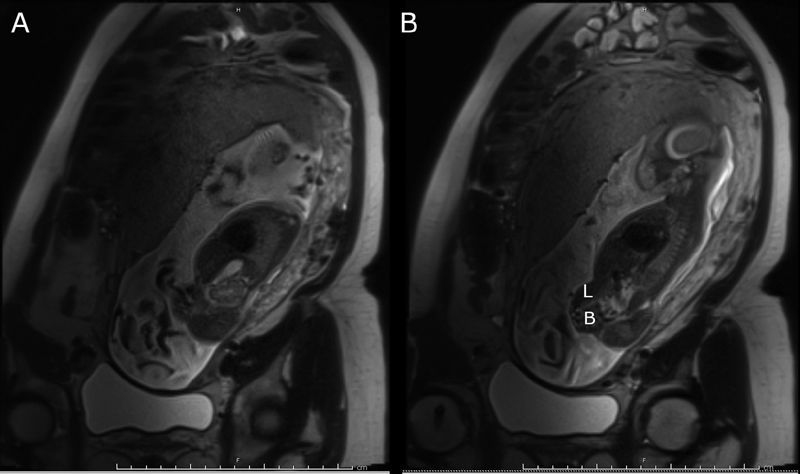
Prenatal MRI demonstrating omphalocele containing liver (L) and bowel (B), absent bladder, and terminal myelocystocele (not pictured on these slices). MRI, magnetic resonance imaging.

The infant's mother is a 28-year-old G3P1, who was referred to our fetal center for evaluation and counseling due to concern for OEIS complex based on prenatal ultrasound. She was initially seen at 22 3/7 weeks of gestation, and underwent magnetic resonance imaging (MRI) and ultrasound, confirming the diagnosis ( Fig. 1 ). At that time, the omphalocele component contained bowel and part of the left lobe of the liver. There was also concern for meconium peritonitis based on areas of calcification scattered over the surface of bowel and liver. A closed lumbosacral neural tube defect (suspected myelocystocele) was visualized, without associated syringohydromyelia. Bilateral clubfeet were noted. Two kidneys were seen, of relatively normal anatomy. Amniotic fluid volume was normal. Repeat ultrasound performed at 31 ^3/7^ weeks showed similar findings. Fetal echocardiography performed at 32 ^4/7^ weeks demonstrated situs solitus without structural defects, valvar, or ventricular dysfunction. The mother established obstetric care at a nearby facility with labor and delivery services and in discussion with urology and general surgery, a plan was established to take the pregnancy to term if possible.

She presented at 33 ^4/7^ weeks in preterm labor with leakage of fluid for over 24 hours. She received one dose of betamethasone and was delivered by cesarean section for breech position. At birth, the infant emerged limp, cyanotic, and apneic with the umbilical cord wrapped around his trunk. Apgar's score at 1 minute was 1, and improved to 9 at 5 minutes, after stimulation and positive pressure ventilation. A Replogle tube and an IV were placed, and he was started on a 10% dextrose solution at a rate of 120 mL/kg/day. Empiric perinatal sepsis coverage was initiated with administration of ampicillin and gentamicin. Birth weight was 2.41 kg.

**Fig. 2:**
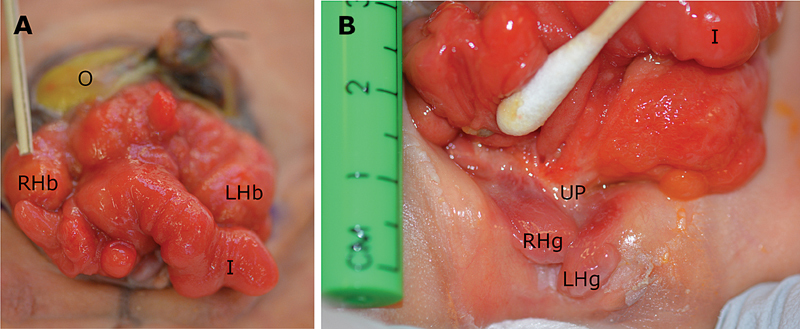
Preoperative abdominal examination. ( **A** ) Visible on initial examination are the omphalocele (O), prolapsed ileum (I), and right and left hemibladders (RHb and LHb). ( **B** ) Reflecting the ileum cranially demonstrates the urethral plate (UP) and bifid penis with two hemiglans (RHg and LHg).

Physical examination was consistent with OEIS ( Fig. 2 ); he had an omphalocele with intact sac containing bowel and minimal liver, imperforate anus, divided bladder plates separated by a cecal plate with prolapsed terminal ileum, as well as bifid glans, penile corpora, and scrota. His exposed bladder and bowel mucosa were protected with an adherent occlusive dressing, and parenteral nutrition was initiated.

Postnatal echocardiogram on day of life (DOL) 2 was notable for a large patent ductus arteriosus (PDA) with bidirectional flow, patent foramen ovale with left-to-right flow, as well as mild right ventricular dilation and hypertrophy. At DOL 6, repeat echocardiography showed that the PDA had become small with continuous left-to-right flow. Perinatal blood cultures remained negative and antibiotics were stopped after 48 hours. Deemed stable for surgery, he was brought to the operating room on DOL 7 for first-stage cloacal exstrophy repair. The planned procedures included excision of the omphalocele sac, separation of the cecal plate and bladder halves, tubularization of the cecal plate, hindgut rescue with end colostomy, and joining of bladder halves.

**Fig. 3:**
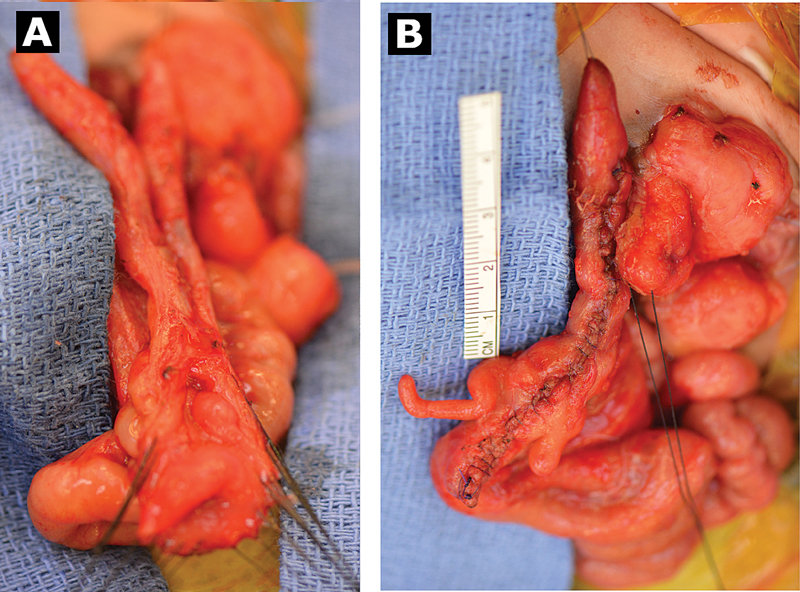
Duplicated hindgut. ( **A** ) Before and ( **B** ) after unification. One hindgut segment was slightly longer than the other resulting in asymmetry of the unified segment.

**Fig. 4:**
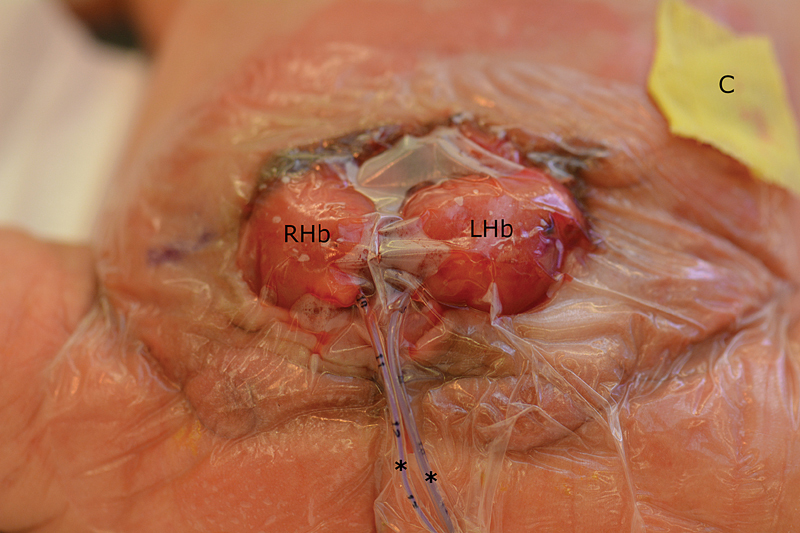
Postoperative appearance of first stage closure. The cecal plate has been tubularized and internalized, allowing unification of the bladder halves (RHb and LHb). Stents (*) are left in each ureteral orifice, and the bladder is covered with an occlusive dressing. A nonadherent petroleum dressing is placed over the end colostomy (C).

In the operating room, two ureteral openings were identified on the bladder plate and ureteral stents were placed. A laparotomy was performed by fully excising the omphalocele sac, allowing better inspection of the peritoneal cavity and understanding of the gastrointestinal anatomy. The terminal ileum was identified by its relation to the cecal plate where it prolapsed externally. Two structures were found descending from the cecal plate, one descended into the pelvis and the second tracked laterally into the left paracolic gutter. They were approximately 8- and 9-cm long. They appeared to emerge from either side of the cecal plate, each with its own orifice, as well as its own mesentery and pair of appendices ( Fig. 3A ). The mesenteries did appear to arise from a common pelvic vascular supply. Consideration was given to separation and end-to-end anastomosis of the individual hindgut segments, but the mesenteries were not mobile enough to allow for this. Therefore, to preserve colonic mucosal surface area, the decision was made to unify them through a side-to-side anastomosis, thereby creating a larger caliber hindgut. The duplicate hindguts were opened longitudinally and underlying mucosa appeared healthy and qualitatively normal on visual inspection. They were aligned by placing 4–0 Vicryl (Ethicon, Johnson and Johnson, Somerville, New Jersey, United States) suture at each apex, and then 5–0 PDS (Ethicon) suture was run continuously, starting posteriorly and then around to the front wall which was contiguous with the open cecal plate. The cecal plate was then tubularized over a Foley's catheter in the usual fashion ( Fig. 3B ). At the end of the case, the unified hindgut appeared healthy and well perfused. The hindgut was then brought out through the abdominal wall and matured as an end colostomy in Brooke's fashion. Postoperative appearance is shown in Fig. 4 .

Postoperatively, the patient was extubated to room air 48 hours after surgery. He remained on parenteral nutrition while awaiting return of bowel function, which occurred on POD 10. Small volume oral breastmilk feeds were initiated and advanced to full volume over several days; feeds were then fortified until he demonstrated appropriate weight gain and was discharged home on POD 30.

Subsequent follow-up has been notable for oral feeding intolerance and failure to thrive. During placement of his gastrostomy tube, he was found to have an odd appearance to the liver. Further imaging and workup demonstrated an extrahepatic portosystemic shunt with associated hyperammonemia. This has been addressed by closure by our interventional radiology colleagues. He subsequently underwent tethered cord release for a terminal myelomeningocele, and second-stage exstrophy repair at 14 months of age including epispadias repair, bilateral osteotomies, creation of penoscrotal hypospadias, and abdominal closure. At 2 years of age, he was at 20th percentile of weight for age.

## Discussion

This case represents a rare association of two complex congenital anomalies. To date, only one prior report exists of a colonic duplication in the setting of OEIS complex. 1 Given the rarity of both of these entities, it is important to consider the challenges each presents and adjust surgical decision making accordingly.

Enteric duplications are uncommon, and hindgut duplications represent a small subset of this anomaly. Resection is generally recommended for both symptom management and risk of malignant transformation. The true risk of malignancy in duplications is unknown as most of them are discovered in childhood and resected. The rate of malignant transformation of persistent duplications is likely small, but cases have been described. 8 9 10 The resection target is clear for cystic lesions, but may be somewhat more difficult to define in tubular duplications if one component is not more clearly aberrant than the other. The duplications can be quite complex; complete duplications and even triplicates have been described, with varying degrees and coincidence with urogenital duplications. 7 11 12

Due to the rarity of both hindgut duplications and OEIS complex, there are no clear guidelines of how to approach and manage this patient's hindgut segments. Current management of OEIS includes preservation of the hindgut in continuity with the rest of the alimentary tract, as an end colostomy to aid with nutrition and fluid management. 5 13 Management options of the duplicated hindgut segments included resection, side-to-side anastomosis, or end-to-end anastomosis. While end-to-end anastomosis would provide the most length, the short respective mesenteries did not allow enough mobility to appropriately anastomose the two segments. By unifying the duplicated hindguts into a single structure through side-to-side anastomosis, nearly all of the absorptive surface area was preserved without placing the mesentery at risk. The increased diameter of the hindgut may also reduce the risk of stomal stenosis, a known complication of end colostomy. With regard to risk of malignancy later in life, the malignancies are reported primarily in patients in whom the duplication is not in continuity with the remainder of the bowel and hence at risk for chronic inflammation. Both the methods of hindgut preservation considered would allow normal enteric flow through the bowel and surveillance via routine colonoscopy. The risk of undetected malignancy, we believe, should remain on par with that of the OIES population without duplications. We project that with better absorptive capacity, the patient will be more likely to form solid stool and may thereby be a candidate for a pull through later in life

The broad manner of presentations of colonic/rectal duplications and associated anomalies of the reproductive and genitourinary system, in combination with their low incidence rate, make them difficult to study in a systematic manner, and thus there is no strong evidence to support optimal management strategies. The only other reported case describes a patient in whom the duplicated hindguts were excised, and the terminal ileum was anastomosed to the perineum. 1 That patient was unable to gain weight and ultimately passed away 2 months after his operation. In our patient, the decision to unify the distinct hindguts into a single lumen was made in an effort to combine the goal of management for both OEIS and alimentary duplications. Although many alimentary tract duplications are excised, the duplicated hindguts in this patient were similar in appearance, so selecting one as “abnormal” for excision would have been an arbitrary choice. Further, the location and management of this duplication allows for ongoing surveillance to evaluate for malignant transformation throughout his life. We believe the risk of malignancy is outweighed by the benefit of optimizing colonic surface area, as OEIS patients are known to experience significant growth morbidity. 14 At this writing, he is currently maintaining his growth curve and developing appropriately. This is a very rare combination of pathologies and this report may help guide others who encounter it and similar pathologies in the future, to best optimize patient outcomes.

## Notes

New Insights and the Importance for the Pediatric Surgeon The coincidence of omphalocele, cloacal exstrophy, imperforate anus, and spinal abnormalities (OEIS complex) and duplicated hindgut is extremely rare and may be managed through hindgut unification, maximizing the preservation of colonic mucosa.
